# Symptomatic Elastofibroma Dorsi Initially Presumed to Be a Subscapular Lipoma: Diagnostic Clues From Dynamic Ultrasonography and Intraoperative Findings

**DOI:** 10.7759/cureus.116909

**Published:** 2026-09-25

**Authors:** Sergio Miguel Suárez Fuentes, Mauro A Cardenas, Andrea Nallely Garza-Cisneros, Luis F Hernández González, Andrea P Rizo navarrete, Karen A Horta Muñoz, Arely S Martínez Roacho, María N Sandoval Arreola, Miroslava A Durán Sandoval

**Affiliations:** 1 General Surgery, Hospital Regional Valentín Gómez Farías, Zapopan, MEX; 2 General Surgery, Presidente Lázaro Cárdenas del Río, Instituto de Seguridad y Servicios Sociales de los Trabajadores del Estado (ISSSTE), Chihuahua, MEX; 3 General Surgery, Hospital Regional de Monterrey, Instituto de Seguridad y Servicios Sociales de los Trabajadores del Estado (ISSSTE), Monterrey, MEX

**Keywords:** chest wall, differential diagnosis, elastofibroma dorsi, soft-tissue tumor, subscapular mass, surgical excision, ultrasonography

## Abstract

Elastofibroma dorsi (ED) is a benign fibroelastic pseudotumor that classically arises in the deep infrascapular region. Although characteristic cross-sectional imaging may establish the diagnosis, a symptomatic subscapular mass may initially be approached as a more common benign soft-tissue lesion when imaging is limited to ultrasonography. We report a 63-year-old woman with a progressively enlarging left subscapular mass associated with persistent pain and discomfort. The initial clinical impression was a probable lipoma, and the patient requested removal because of ongoing symptoms. Targeted ultrasonography demonstrated a 35.3 × 8 mm heterogeneous, irregularly marginated, avascular lesion closely related to a rib. Its apparent dimensions varied with movement, and forward positioning of the arm was required to displace the scapula and obtain an adequate acoustic window. Because the lesion was readily localized and excision had already been selected for progressive growth and symptoms, computed tomography or magnetic resonance imaging was not obtained. At surgery, the lesion was firm, fibrous, poorly circumscribed, and tightly adherent to the deep muscular and costal planes rather than lipomatous, prompting intraoperative suspicion of ED. Muscle-sparing en bloc excision was completed without pleural entry or apparent intraoperative complication. Histopathology showed dense collagen, abnormal thick fragmented elastic fibers, and interspersed mature adipose tissue without atypia, mitotic activity, necrosis, or malignancy, confirming ED. At the documented postoperative follow-up visit, the patient had no pain, seroma, infection, or clinical evidence of recurrence. This case emphasizes that a growing, painful deep subscapular mass clinically presumed to be a lipoma warrants attention to scapulothoracic anatomy and dynamic sonographic behavior. When ultrasonographic findings are non-specific, cross-sectional imaging may improve preoperative recognition, while the characteristic intraoperative anatomy can provide an important diagnostic clue.

## Introduction

Elastofibroma dorsi (ED) is a benign fibroelastic pseudotumor that typically arises deep to the inferior angle of the scapula, in the scapulothoracic plane adjacent to the posterior chest wall and lower ribs, frequently deep to the latissimus dorsi and in proximity to the serratus anterior muscle. Repetitive scapulothoracic friction has been proposed as a contributory mechanism, although its etiology is not fully established [1]. It occurs predominantly in middle-aged and older adults, particularly women, and subclinical lesions are more common than clinically recognized cases [1-3]. Histologically, ED is characterized by dense collagen, abnormal elastic fibers, and entrapped mature adipose tissue. This mixture produces the characteristic alternating fibrous and fatty architecture described on ultrasonography, computed tomography (CT), and magnetic resonance imaging (MRI) [1,4-7].

The clinical spectrum ranges from an incidental, asymptomatic mass to progressive swelling, discomfort, pain, scapular snapping, or limitation of shoulder motion [8-15]. Bilateral lesions have also been reported, although their frequency varies considerably across clinical and imaging populations [2,10-14]. Typical imaging in an appropriate anatomical location may be sufficiently characteristic to avoid biopsy or surgery in asymptomatic patients, whereas excision is generally reserved for symptomatic, enlarging, diagnostically uncertain, or otherwise clinically significant lesions [1,8-15]. The diagnostic challenge arises when the lesion is clinically presumed to be a common benign mass and preoperative imaging is non-specific. We present an ED deep to the left inferior scapular angle that was initially considered a lipoma; its distinctive educational feature is the combination of arm position-dependent ultrasonographic visualization and recognition of the non-lipomatous lesion during surgery before histopathologic confirmation.

## Case presentation

A 63-year-old woman was evaluated for a palpable mass deep to the inferior angle of the left scapula over the posterior thoracic wall. She had first noticed the lesion approximately 18 months earlier when local pain began. The pain was continuous and worsened with arm movement or pressure when resting her back against a surface. She reported slow, progressive enlargement without a sudden increase in growth. There was no relevant history of regional trauma, previous treatment for the mass, or other pertinent medical or surgical history. The initial clinical working diagnosis was a probable lipoma. Because the pain persisted and the lesion continued to enlarge, she requested removal, and elective surgical excision was planned.

On examination, a soft, mobile mass was palpable beneath the left inferior scapular angle and was tender to palpation. The prominence became more evident with scapular movement; shoulder range of motion was preserved. No formal clinical measurement of the palpable mass was recorded. Overlying skin changes and scapular snapping were not specifically documented.

Targeted soft tissue ultrasonography was performed with a high-frequency linear transducer using B-mode and color Doppler imaging. A 35.3 × 8 mm heterogeneous sonographically visualized component with irregular margins and no detectable color Doppler vascularity was identified in the left infrascapular/posterior thoracic region, deep to the inferior scapular angle. The recorded dimensions describe what was measurable through the available acoustic window; whether they represented the entire lesion could not be established. It was closely related to an underlying rib, and its apparent dimensions changed with movement. Forward positioning of the arm displaced the scapula sufficiently to obtain an acoustic window. The exact transducer frequency, three orthogonal measurements, and quantitative dimensions at successive arm positions were not recorded; a characteristic fasciculated pattern was not established by the available examination. The ultrasonographic appearance is shown in Figure 1.

**Figure 1:**
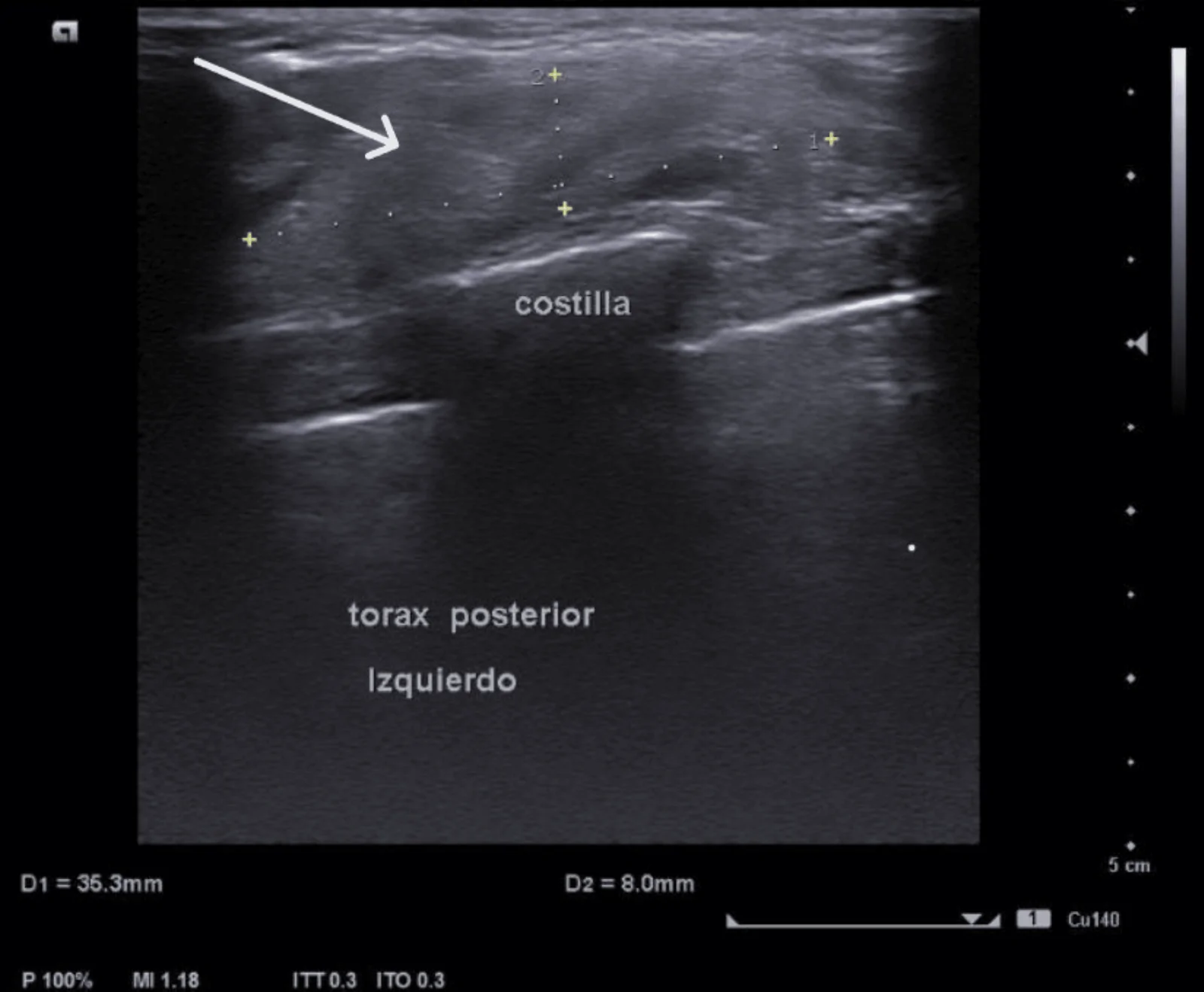
Ultrasonographic appearance of the left subscapular elastofibroma dorsi Targeted ultrasonography of the left posterior thoracic/subscapular region showing a heterogeneous, well-visualized soft-tissue lesion delineated by the sonographic calipers and closely related to the underlying rib. The lesion demonstrated no detectable internal vascularity on color Doppler examination. Forward positioning of the left arm was required to displace the scapula and obtain an adequate acoustic window.

The ultrasound report recommended clinical correlation and consideration of further imaging. Although CT or MRI could have provided additional tissue characterization, the lesion was localized on targeted ultrasound and the decision to excise it had already been made because of progressive enlargement, persistent symptoms, and the patient's preference for removal. Therefore, no cross-sectional imaging was obtained before surgery. The clinical sequence is summarized in Table 1.

**Table 1:** Clinical timeline

Relative interval	Documented event
Approximately 18 months before surgery	Onset of local pain and recognition of the infrascapular mass; subsequently, slow progressive enlargement
Preoperative period	Clinical assessment and targeted ultrasound; the lesion was clinically presumed to be a lipoma; symptomatic excision selected
Surgery, week 0	Muscle-sparing en bloc excision; intraoperative features suggested elastofibroma dorsi
Postoperative week 2	First postoperative assessment; uncomplicated clinical recovery
Postoperative week 3	Second assessment and pathology report confirming elastofibroma dorsi; pain 0/10; discharge from routine follow-up

Under balanced general anesthesia with endotracheal intubation, the patient was placed prone with appropriate padding and exposure of the region deep to the left inferior scapular angle. The lesion was localized by directed palpation. An approximately 6 cm incision was made along the inferior border of the left scapula, following the local skin tension lines. Dissection proceeded through the subcutaneous tissue with electrocautery hemostasis until the superficial muscular fascia was identified. The fibers of the latissimus dorsi were separated longitudinally to avoid unnecessary transection and permit access to the deep subscapular plane.

Deep dissection beneath the inferior scapular angle revealed a solid, poorly circumscribed, firm, predominantly fibrous lesion whose macroscopic appearance was markedly different from adipose tissue (Figure 2).

**Figure 2:**
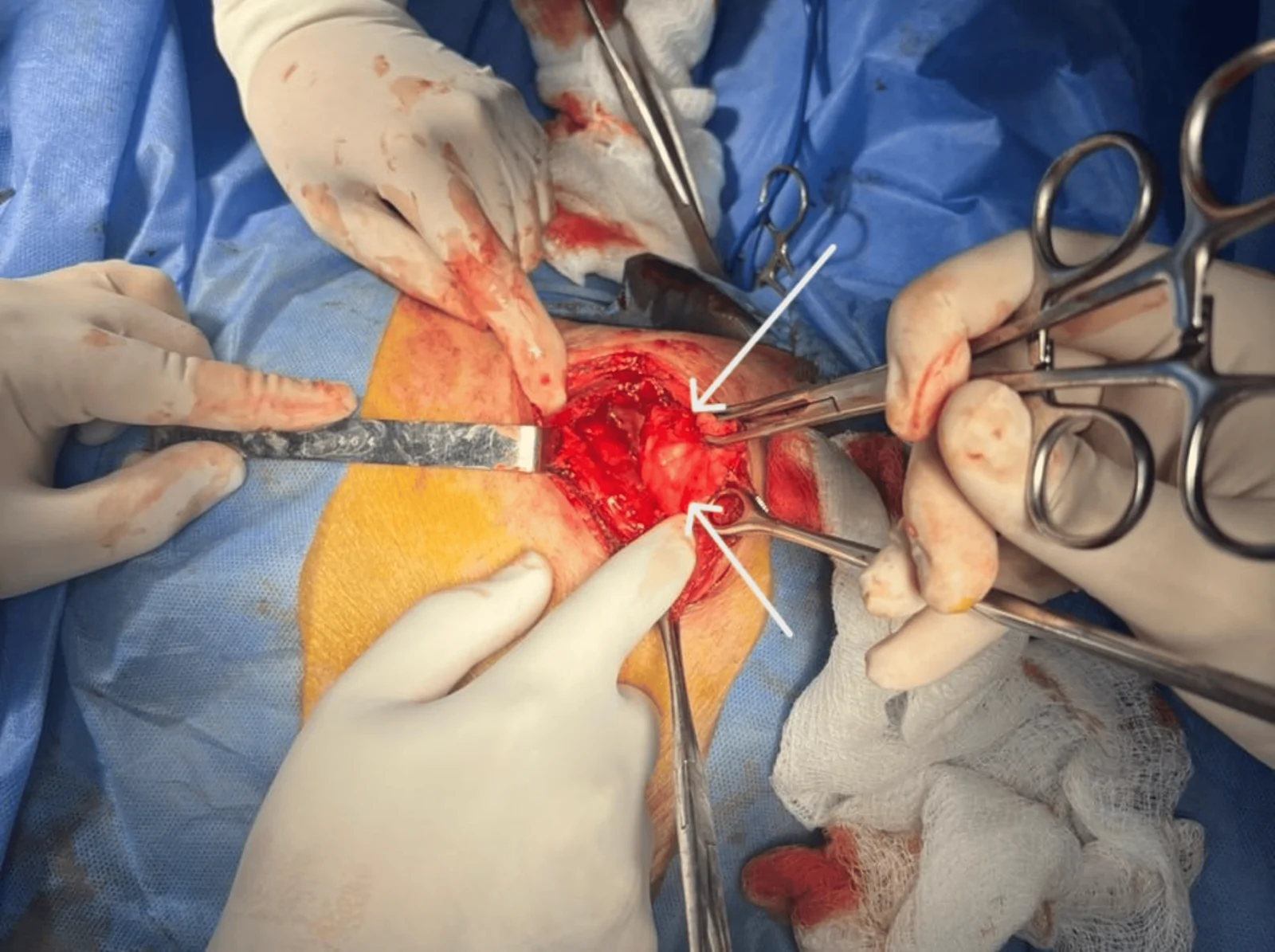
Intraoperative identification of the left subscapular lesion Initial intraoperative identification of the left subscapular lesion after a muscle-sparing approach. The lesion appeared firm and fibrous rather than lipomatous, prompting intraoperative suspicion of elastofibroma dorsi.

At that point, ED became the leading intraoperative diagnosis. The lesion was progressively mobilized using blunt dissection and electrocautery. It was tightly adherent to the surrounding deep muscular planes and intimately related to the chest wall and costal surface. Circumferential dissection released the lesion while preserving the latissimus dorsi and deeper chest wall musculature (Figure 3).

**Figure 3:**
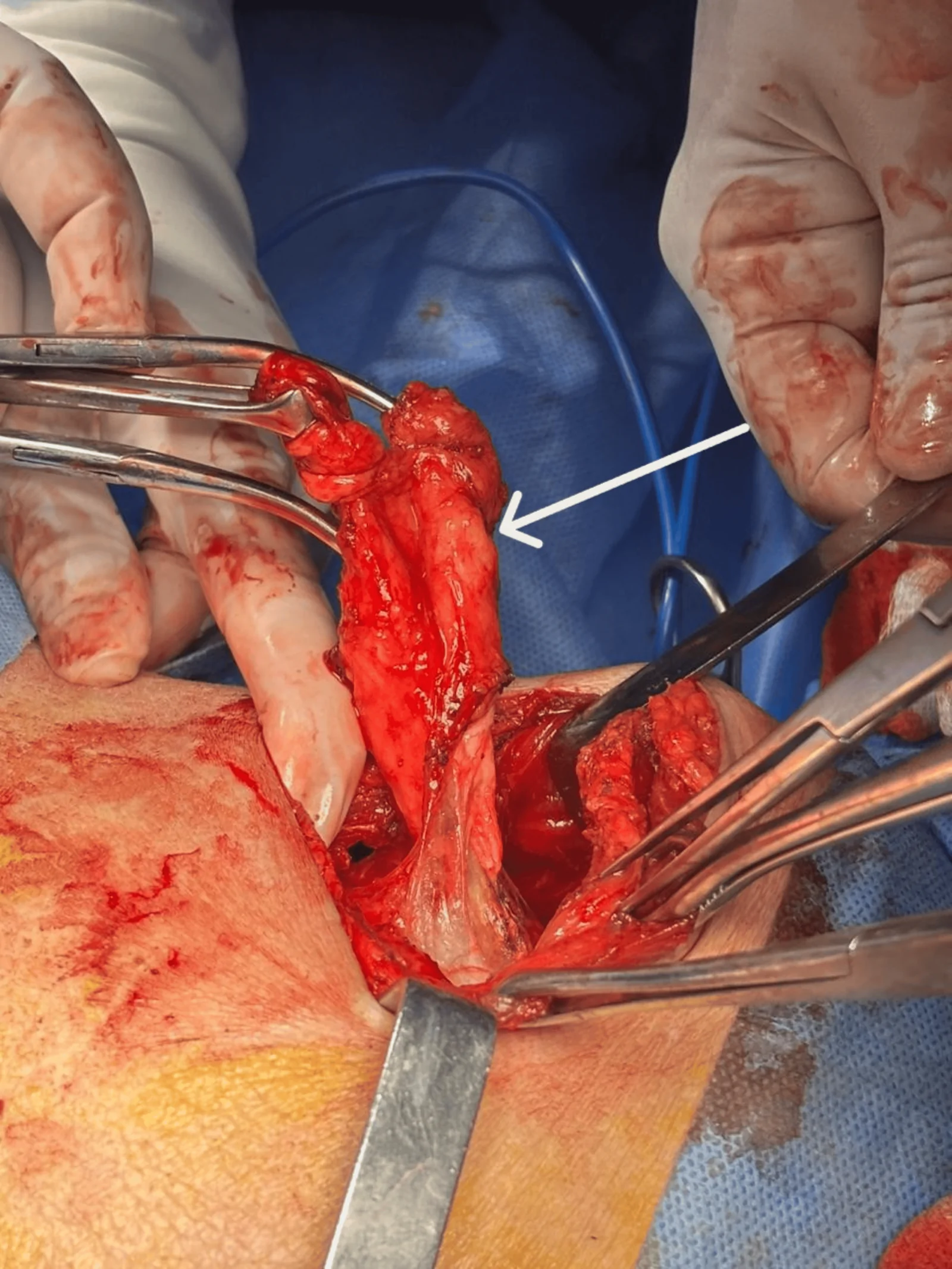
Advanced dissection of the elastofibroma dorsi from the deep chest wall planes Advanced circumferential mobilization of the lesion from the surrounding muscular and costal planes. The mass was tightly adherent to the deep tissues and was carefully separated while preserving the adjacent musculature and avoiding pleural entry.

The deep surface was separated carefully from the rib cage without pleural entry or evident structural injury to the chest wall. Residual fibrovascular tissue was controlled with polyglactin ligature and divided, permitting en bloc removal. After irrigation, meticulous hemostasis was confirmed, and the wound was closed anatomically in layers. No apparent intraoperative complication occurred. The excised lesion was reported to measure 42.4 × 10 mm in two dimensions; a third dimension, specimen weight, and formal gross margin measurements were unavailable.

Histopathologic examination established the diagnosis of ED. Hematoxylin and eosin (H&E)-stained sections showed a poorly demarcated, hypocellular lesion with abundant dense collagen bundles containing numerous abnormal eosinophilic elastic fibers that were thick, wavy, fragmented, and irregularly contoured, including globular or beaded forms. Mature adipose tissue was interspersed throughout the fibroelastic tissue. The spindle cell component showed small, uniform nuclei without significant cytologic atypia or pleomorphism. No identifiable mitotic activity, tumor necrosis, histologic evidence of malignancy, or malignant transformation was present (Figure 4). A specific elastic fiber stain was not documented, and microscopic margin status was unavailable. The gross resected specimen is shown in Figure 5.

**Figure 4:**
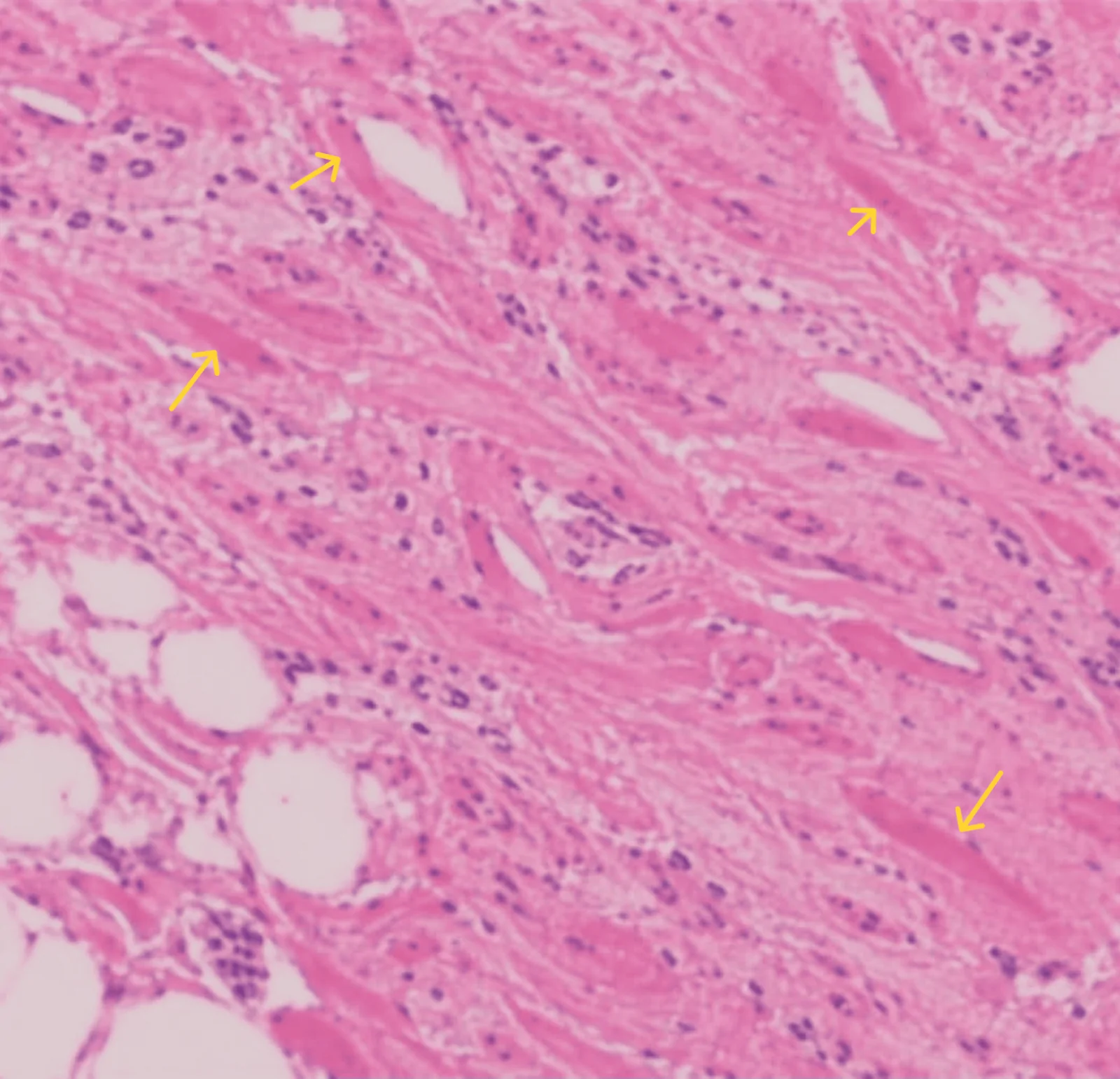
Histopathologic features of elastofibroma dorsi Histopathologic examination of the resected lesion showing a hypocellular fibroelastic proliferation composed of abundant dense collagen bundles interspersed with mature adipose tissue. Numerous abnormal eosinophilic elastic fibers are present within the collagenous stroma, displaying thickened, irregular, fragmented, and focally beaded or globular contours (arrows). Scattered bland spindle cells with small uniform nuclei are identified without significant cytologic atypia. No mitotic activity, tumor necrosis, or malignant features are evident. The findings are characteristic of elastofibroma dorsi.

**Figure 5:**
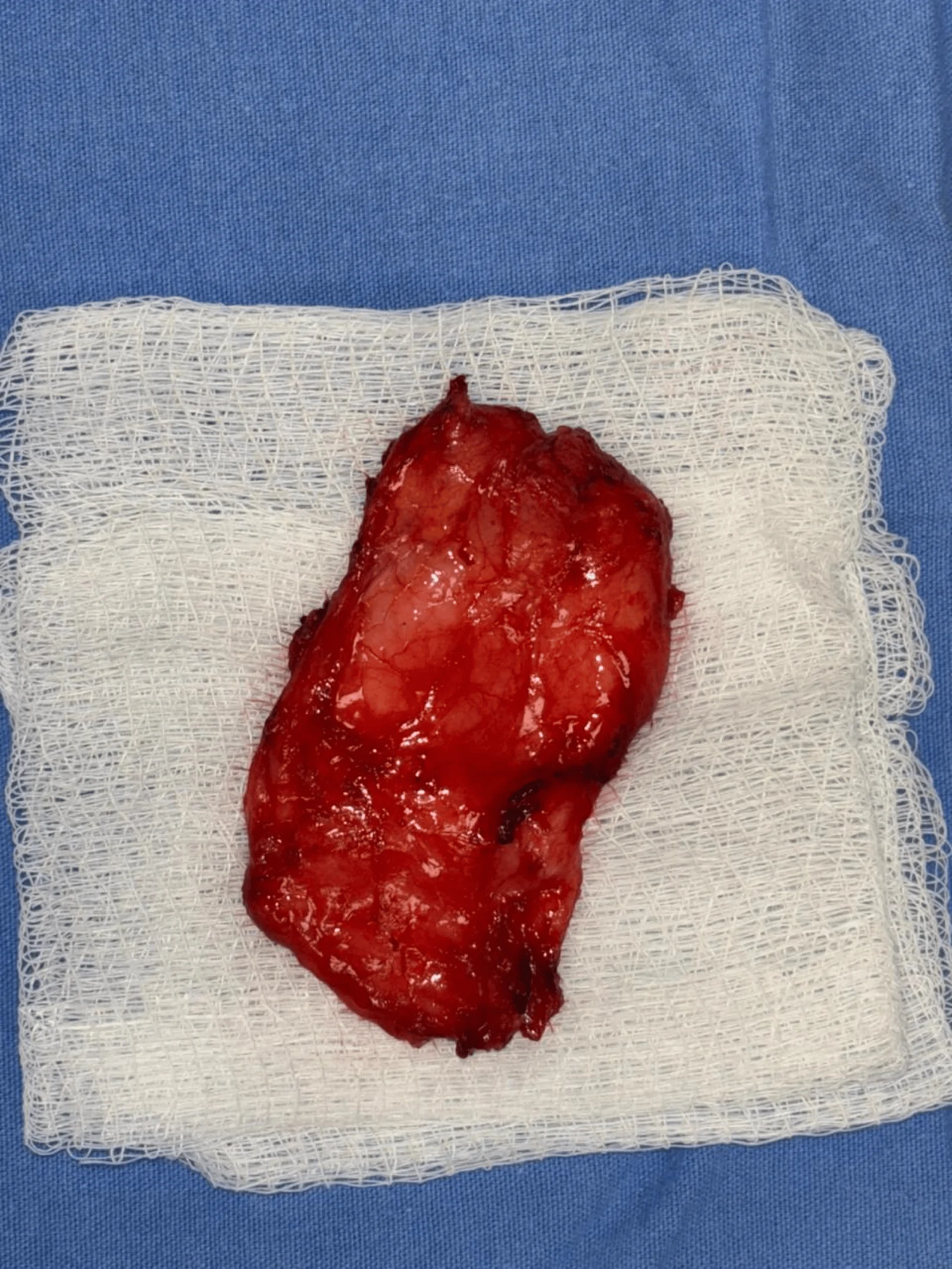
Gross appearance of the resected elastofibroma dorsi Gross appearance of the resected left infrascapular specimen. The lesion showed a predominantly fibrous appearance; the reported two-dimensional measurement was 42.4 × 10 mm. A third dimension and specimen weight were unavailable. Histopathologic examination confirmed elastofibroma dorsi.

Postoperative evolution was favorable. The patient attended two scheduled postoperative visits, at two and three weeks after surgery. No pain, seroma, surgical site infection, or clinically apparent recurrence was identified at either assessment. By the three-week visit, the patient reported no pain or discomfort (visual analog scale, 0/10), was recovering appropriately, and was discharged from routine postoperative follow-up. Histopathology was reported approximately three weeks after surgery. No later follow-up examination was available. Written informed consent for treatment and for open-access publication of the clinical information and accompanying images was obtained from the patient.

## Discussion

This case illustrates a practical diagnostic problem rather than an unusual histologic variant: ED may be clinically mistaken for a lipoma when a mass deep to the inferior scapular angle is evaluated without characteristic cross-sectional imaging. The patient’s age, sex, and infrascapular location deep to the inferior scapular angle were typical for ED. Contemporary surgical series continue to show a strong female predominance, frequent symptoms among patients selected for resection, and a substantial rate of bilateral disease [11-14]. The key reason to recognize ED is not malignant potential but to select appropriate imaging and treatment while avoiding unnecessary diagnostic escalation for a benign lesion.

Symptoms provide an important distinction between lesions that can be observed and those for which surgery is reasonable. Pain, discomfort, scapular snapping, and functional symptoms are well described, and surgery has traditionally been reserved for symptomatic or clinically significant lesions [8,10-15]. In the present patient, 18 months of slow enlargement together with continuous pain exacerbated by arm movement or pressure on the back provided a direct clinical indication for excision independent of the eventual histologic diagnosis. Thus, the main missed opportunity was preoperative characterization rather than the decision to treat.

Ultrasonography can provide highly useful information in the ED, particularly when the lesion demonstrates the classic fasciculated pattern of alternating hypoechoic and echogenic linear components parallel to the chest wall [4,7]. However, sonographic appearances are not uniform. Battaglia et al. described both characteristic and non-specific ultrasonographic patterns, supporting CT or MRI for non-fasciculated lesions and in selected surgical candidates [7]. Positioning is also important because the scapula may conceal the mass. Dalal et al. reported that an ED became sonographically apparent only after arm repositioning displaced it from beneath the scapula [5]. The need to bring our patient’s arm forward to expose the lesion, together with the change in apparent dimensions during movement, is therefore a meaningful anatomical clue rather than merely a technical detail. In the present examination, the change with arm positioning was recorded qualitatively rather than through serial numerical measurements; the ultrasound data do not establish that the classic fasciculated pattern was absent.

In retrospect, the heterogeneous non-lipomatous appearance, irregular margins, close relationship to a rib, and dynamic behavior on ultrasonography could have prompted CT or MRI before surgery. Cross-sectional imaging typically shows a lenticular, poorly circumscribed mass with attenuation or signal intensity similar to skeletal muscle interlaced with streaks of fat [6,7]. In this case, CT or MRI was not obtained because the lesion was readily localized by ultrasound and symptom-driven excision had already been selected. This approach did not prevent definitive treatment, but the experience highlights how cross-sectional imaging could have improved diagnostic confidence, operative planning, and preoperative counseling. The 35.3 × 8 mm sonographic dimensions describe what was measurable through the available acoustic window; completeness of lesion visualization was uncertain, whereas the resected specimen was reported to measure 42.4 × 10 mm; without a third dimension or volumetric assessment, these two-dimensional values are not directly interchangeable.

The operative findings were highly concordant with ED. Instead of an encapsulated adipose mass, the surgeons encountered a firm, fibrous, poorly defined lesion adherent to the deep scapulothoracic and costal planes. Similar chest wall fixation and indistinct margins have been described in surgical series and reflect the lesion’s intermixture with adjacent fibro-fatty tissue rather than malignant invasion [10-12,15]. Marginal en bloc excision through a muscle-sparing approach is generally adequate when surgery is indicated [10-14]. Seroma and hematoma are among the most frequently reported postoperative complications, making meticulous hemostasis and dead space management important [11-15]. At postoperative assessments at two and three weeks, neither complication was evident, and the preoperative pain had resolved (0/10 at the final visit).

Histopathology provided definitive confirmation. The combination of dense collagen, coarse fragmented elastic fibers, mature adipose tissue, and bland spindle cells is characteristic of ED [1,15]. The absence of atypia, mitotic activity, and necrosis supported the benign nature of the lesion. The firm fibrous consistency encountered intraoperatively is readily explained by this collagen-rich fibroelastic composition and helps distinguish the gross appearance from that of a conventional lipoma. Figure 4 documents H&E morphology; because the original image lacks an authenticated microscope scale and magnification record, numerical magnification or a scale bar cannot be assigned reliably.

The educational contribution of this case differs from previously published Cureus reports. Fabien et al. described symptomatic ED diagnosed on CT before resection [8], whereas Pham et al. reported an asymptomatic lesion investigated because of concern for malignancy in an oncologic patient and ultimately managed non-operatively [9]. In contrast, the present case followed an ultrasound-only preoperative pathway: a growing painful mass was clinically presumed to be a lipoma, arm positioning was required for sonographic visualization, and the diagnosis became apparent only when the intraoperative fibrous anatomy contradicted the expected lipomatous lesion. This sequence emphasizes the value of integrating dynamic ultrasound behavior, scapulothoracic anatomy, and operative findings when the preoperative diagnosis remains uncertain.

This report has limitations. CT and MRI were not obtained, so the case cannot demonstrate the classic cross-sectional imaging appearance or determine whether those modalities would have established the diagnosis prospectively. The two- and three-week postoperative assessments confirmed an uncomplicated early clinical course, but long-term follow-up was not available to assess late recurrence. Exact transducer frequency, serial position-dependent ultrasound measurements, a third specimen dimension, weight, formal margin status, and calibrated histologic magnification were unavailable. These limitations do not affect the histopathologic diagnosis but should temper conclusions about imaging performance and long-term outcome.

## Conclusions

In a patient with a slowly enlarging, painful mass deep to the inferior scapular angle, ED should remain in the differential diagnosis even if initial palpation suggests a lipoma. Arm position-dependent visualization and a heterogeneous lesion abutting a rib on ultrasonography are anatomical clues, not stand-alone diagnostic criteria. When ultrasonographic morphology is non-specific, CT or MRI can improve preoperative characterization and operative planning. In symptomatic patients selected for excision, a firm, poorly circumscribed fibroelastic lesion adherent to the chest wall should prompt intraoperative consideration of ED; histopathology confirms the diagnosis. The present case documents an uncomplicated early postoperative course but does not establish long-term recurrence outcomes.
